# Mesenchymal Progenitor Cells and Their Orthopedic Applications: Forging a Path towards Clinical Trials

**DOI:** 10.4061/2010/519028

**Published:** 2010-12-16

**Authors:** Deana S. Shenaq, Farbod Rastegar, Djuro Petkovic, Bing-Qiang Zhang, Bai-Cheng He, Liang Chen, Guo-Wei Zuo, Qing Luo, Qiong Shi, Eric R. Wagner, Enyi Huang, Yanhong Gao, Jian-Li Gao, Stephanie H. Kim, Ke Yang, Yang Bi, Yuxi Su, Gaohui Zhu, Jinyong Luo, Xiaoji Luo, Jiaqiang Qin, Russell R. Reid, Hue H. Luu, Rex C. Haydon, Tong-Chuan He

**Affiliations:** ^1^Molecular Oncology Laboratory, Department of Surgery, The University of Chicago Medical Center, 5841 South Maryland Avenue, MC3079, Chicago, IL 60637, USA; ^2^Key Laboratory of Diagnostic Medicine Designated by Chinese Ministry of Education, The Affiliated Hospitals of Chongqing Medical University, Chongqing 400016, China; ^3^Stem Cell Biology and Therapy Laboratory, The Children's Hospital of Chongqing Medical University, Chongqing 400014, China; ^4^School of Bioengineering, Chongqing University, Chongqing 400030, China; ^5^Department of Geriatrics, Xinhua Hospital of Shanghai Jiatong University, Shanghai 400092, China; ^6^Department of Cell Biology, The Third Military Medical University, Chongqing 400042, China

## Abstract

Mesenchymal progenitor cells (MPCs) are nonhematopoietic multipotent cells capable of differentiating into mesenchymal and nonmesenchymal lineages. While they can be isolated from various tissues, MPCs isolated from the bone marrow are best characterized. These cells represent a subset of bone marrow stromal cells (BMSCs) which, in addition to their differentiation potential, are critical in supporting proliferation and differentiation of hematopoietic cells. They are of clinical interest because they can be easily isolated from bone marrow aspirates and expanded in vitro with minimal donor site morbidity. The BMSCs are also capable of altering disease pathophysiology by secreting modulating factors in a paracrine manner. Thus, engineering such cells to maximize therapeutic potential has been the focus of cell/gene therapy to date. Here, we discuss the path towards the development of clinical trials utilizing BMSCs for orthopaedic applications. Specifically, we will review the use of BMSCs in repairing critical-sized defects, fracture nonunions, cartilage and tendon injuries, as well as in metabolic bone diseases and osteonecrosis. A review of www.ClinicalTrials.gov of the United States National Institute of Health was performed, and ongoing clinical trials will be discussed in addition to the sentinel preclinical studies that paved the way for human investigations.

## 1. Introduction: What Are Stem Cells?

The popularity of stem cells in the clinical arena has significantly increased, given the rapid improvement in our understanding of their biology. Classically, stem cells are defined by their capacity to retain an undifferentiated state for a prolonged period while retaining the potential to differentiate along one lineage (unipotent), multiple lineages (multipotent), or into all three germ layers (pluripotent) [1]. These cells can be broadly categorized into two major classes: embryonic and adult stem cells. Embryonic stem cells (ESCs), isolated from the inner cell mass of the blastocyst, are pluripotent cells with the potential of differentiating into tissues from all three germ layers [2, 3]. While ESCs have significant regeneration capacity, their clinical application has been limited as a result of multiple factors including: (1) a propensity to form teratomas, (2) ethical concerns with isolation, (3) rejection by the host immune system after transplantation, and (4) the use of a feeder layer to retain an undifferentiated state in vitro [4–6]. Recently discovered, another source of pluripotent stem cells are induced pluripotent stem (iPS) cells, derived from somatic cells treated with few defined factors [7–11]. While iPS cell-based therapy has the potential to revolutionize the field of regenerative medicine, many obstacles must be overcome before their clinical application can be realized [12].

Furthermore, naturally occurring adult stem cells have also been identified and categorized into either hematopoietic stem cells (HSCs), a source of various hematopoietic cell lineages, and nonhematopoietic stem cells, which can give rise to cells of mesenchymal origin [13]. Many reports have suggested that these nonhematopoietic stem cells, also known as mesenchymal progenitor cells (MPCs), can be isolated from various tissues including blood, adipose, skin, mandible, trabecular bone, fetal blood, liver, lung, and even the umbilical cord and placenta [14, 15]. Upon harvest, these cells can be expanded in vitro with high efficiency without sacrificing differentiation capacity [16–20]. While these multipotent progenitor cells share many similar characteristics, they can be differentiated based on their expression profile and differentiation propensity along various lineages [21]. Amongst the various sources, MPCs isolated from the bone marrow, a subset of Bone Marrow Stromal Cells (BMSCs) are considered to have the greatest potential for multilineage differentiation and have been the most characterized [22, 23]. 

 BMSCs were initially described by Friedenstein and colleagues more than 40 years ago as adherent cells, with a fibroblast-like appearance capable of differentiating into osteoblasts, chondroblasts, adipocytes, and tenocytes [22, 24, 25]. Unlike ESCs, BMSCs provide the flexibility of autologous transplantation, circumventing ethical concerns or immunologic rejection [26].These cells also play a sentinel role in proliferation and differentiation of hematopoietic cells [27]. Mankani et al. illustrated that the formation of both hematopoiesis and mature bone organ is correlated with the high local density of BMSCs [28]. Additionally, BMSCs are considered to be immune privileged and have the capacity for allogenic transplantation a property that has been used in the clinical setting for the treatment of various autoimmune diseases [29–31]. While many studies have suggested that MPCs are immunoprivileged and do not undergo rejection, others have cast doubt on this notion, showing that in certain scenarios, MPCs induce immune rejection [32]. More investigations should be conducted to provide further insight into the specific interaction between these progenitor cells and the host immune system. 

Considerable effort has been put forth to identify specific surface markers that characterize MPCs, yet disagreement within the literature has prevented the creation of definitive standards. The minimal criteria identified by the International Society for Cellular Therapy for identifying BMSCs requires the cells: (1) to be plastic adherent while maintained in cell culture, (2) to express CD73, CD90, and CD 105 and lack expression of CD11b, CD14, CD19, CD34, CD45, CD79-alpha, and HLA-DR, and (3) to differentiate into osteoblasts, adipocytes, and chondroblasts in vitro [33]. Additional studies have also suggested that CD146 is considered an important marker of bone marrow progenitor cells [34, 35]. These guidelines were set in place to enable a unified approach for comparison amongst different studies. 

Bone marrow is generally considered a milieu plentiful for various cell types, collectively referred to as stromal cells. Amongst these, the multipotent subset of BMSCs comprises a small fraction (<0.01) [36], yet despite their small numbers, the relative ease with which BMSCs can be harvested has propelled their experimental use. Researchers have pioneered the creation of stable animal models aimed at mimicking human conditions to study the therapeutic capacity of these bone marrow-derived cells [37]. Because of their ubiquity, tolerance of expansion, paracrine capabilities, and multipotency, the potential for clinical applications of BMSCs in the orthopaedic realm is countless.

In this paper, we will focus on the development of human clinical trials utilizing BMSCs for orthopaedic applications. The path towards the creation of such trials beginning from sentinel animal investigations will be highlighted. Specifically, the progenitor subset of BMSCs used in the context of critical-sized defects, fracture nonunions, cartilage and tendon repair, metabolic bone disease, and osteonecrosis will be discussed. A review of www.ClinicalTrials.gov of the United States National Institute of Health was performed to underscore the status of ongoing clinical trials.

## 2. Modes of Use and Preparation

As previously mentioned, MPCs have been successfully harvested from a variety of tissues; however, most clinical trials utilize BMSCs for therapeutic applications. These cells are harvested from the iliac crest and expanded ex vivo in supplemental media. Cell expansion is currently a time-consuming process, generally requiring 3–5 weeks to obtain sufficient number for therapeutic application [38]. This paper will touch upon the different strategies for MPCs preparation and use within the clinical arena, and therefore, a prior overview of such approaches should be outlined. As the most commonly used cell type for clinical application, BMSCs can be administered through either autologous or allogenic transplantation. In clinical trials, fresh autologous BM and passaged BMSCs have been utilized for cellular therapy [39]. Freshly harvested aspirates can further undergo centrifugation to concentrate BMSCs prior to percutaneous or intravenous injection [40–42]. When expanded ex vivo, various cell-specific characteristics are utilized to enrich progenitor cells and separate them from other cells in the bone marrow. One such characteristic that enables their selection is BMSC preferential adherence to the plastic surface of the culture dish [43, 44]. Immunoselection for previously discussed MPC markers also enables further identification [45–47]. After expansion and selection, the harvested cells can be utilized for various therapeutic applications. 

Subsequent to ex vivo expansion, BMSCs can be cultured in the presence of a scaffold-enabling colonization and cell differentiation prior to material grafting at the affected site [48]. Preconditioning of the graft-scaffold composite can be performed in vivo. For example, the composite can be first inserted into a heterotopic, richly vascularized area in order to stimulate angiogenesis within the intended implant. After adequate growth, the newly vascularized tissue can be excised and inserted into the desired site, thus increasing the chance for cell survival [49]. Mankani and colleagues impregnated BMSCs into a hydroxyapatitie/tricalcium phosphate (HA/TCP) matrix and subsequently observed the generation of mature bone with similar histologic and mechanical properties to the bone formed with standard transplant techniques in animal models [50]. The above-mentioned modes of administration are only a few of the many methods that various clinical trials have utilized to provide innovative therapeutic strategies for many diverse clinical applications.

## 3. Critical-Sized Bone Defects

Critical-sized defects are osseous defects beyond certain size where complete healing will not occur during the lifetime of the organism. Sixty years ago, the idea of autografting arose as a solution for repairing large defects that would otherwise not bridge in the normal environment. It was clear then that tissue progenitors were necessary for effective defect repair. Classically, grafting involves the harvest of unaffected pieces of tissue from remote sites and their implantation into affected areas. Yet, autograft use is limited by the lack of sufficient donor tissue to fill large defects and the morbidity afflicted to the donor site. These clinical drawbacks have led to the search for cells that could be induced to differentiate and grow into the desired tissue type [51]. 

The concept of using cells to repair tissues is continually evolving. The idea that MPCs possess the capacity for bone regeneration in vitro and ectopically in vivo has been tested and reaffirmed in various studies [52]. Promising results spurned the development of animal models as building blocks for the eventual clinical use of stem cells in humans. These models can mimic (1) normal fracture healing, (2) critical-sized segmental defects, and (3) non-critical-sized defects, as in the case where healing is prevented by fracture nonunions [51]. A variety of animal models including mice, rats, dogs, sheep, and goats have been utilized to study the role of MPCs in promoting repair in critical-sized defect. These studies have also investigated critical size defects of different anatomical locations including: the femur, tibia, metatarsal, mandible, and calvaria [53]. 

In a pilot study performed on a sheep model, Kon et al. reported the use of autologous bone marrow stromal cells in conjunction with hydroxyapatite ceramic (HAC) carriers and demonstrated faster bone repair in the BMSC-treated group as compared to HAC alone [54]. Potentially, this combination could be used clinically for the treatment of significant long bone defects. In 2003, Arinzeh et al. further expanded upon earlier trials and found that autologous and allogeneic MPCs could repair critical-sized bone defects in the canine model without immunosuppressive treatment [55]. Kuznetsov and colleagues demonstrated that BMSCs have the capacity to provide long-term bone augmentation of the mandible [56]. In an earlier study, a similar group concluded that BMSCs in conjunction with HA/TCP can successfully close craniofacial bony defects in a mouse model [57]. Biomechanically, the newly formed bone demonstrates similar properties as the normal bone surrounding the defect site [58]. Encouraging results from these animal studies and others have paved the way for investigations involving humans.

Quarto et al. provide one of the first clinical reports involving the repair of segmental diaphyseal defects in 4 patients using culture-enriched ex vivo expanded progenitor cells [59]. Autologous bone marrow stromal cells were placed on macroporous hydroxyapatite (HA) scaffolds, with their size and shape dependent on the characteristic of the specific bony defects. These grafts were stabilized via external fixation. Integration at the bone-implant interface was observed one month postoperatively and complete consolidation was evident after 5–7 months. All patients recovered limb function with no complications associated with the implants. At last followup (6 to 7 years postoperatively in patients 1 to 3), a good integration of the implants was maintained without evidence of late fracture [60]. 

Ohgushi et al. have expounded upon earlier studies by pioneering the production of bone matrix ex vivo. After expanding marrow-derived MPCs, the investigators then subcultured the osteoprogenitor cells to allow for the fabrication of osteoblasts/bone matrix onto various substrata. The in vitro cultured regenerative bone was then delivered to hospitals for surgical use. Care was taken to assure the risk of bacterial/fungal contamination is minimized. Since 2002, Ohgushi and colleagues have been utilizing these techniques clinically for the treatment of chronic skeletal diseases (i.e., OA and benign bone tumors) [61].

The use of scaffolds or inert materials impregnated with MPCs to generate three-dimensional implants is a branch of tissue engineering which is rapidly growing. By utilizing scaffolds, researchers attempt to create an in vivo environment that favors the development of the desired tissues for implantation. Seeding these composites with the progenitor cells creates a potential for increased bony regeneration because it enhances the repair process by supplying progenitors that secrete factors. Perhaps one of the greatest examples of this mode of tissue engineering for bony regeneration is the case report by Vacanti et al., where the authors replaced the avulsed phalanx of a 36-year-old man with periosteal progenitor cells harvested from the distal radius seeded onto a natural coral (porous HA) scaffold. This procedure resulted in the functional restoration of a biomechanically sound thumb of normal length, without the comorbidity associated with harvesting bone grafts [62].

Promising preclinical studies and case reports have provided support for the therapeutic role of culture-expanded osteoprogenitor cells and their application in conjunction with different scaffolds for healing of long bone defects in clinical trials. Hoping to take advantage of this tissue-engineering approach, Emory University gained approval in March 2009 to conduct a Phase II/III randomized, single-blinded control trial with 50 participants utilizing the allograft substance Trinity in the repair of bony deficits in patients with benign disease (NCT00851162). Trinity is currently FDA approved for use in traumatic bony defects including the spine. MPCs, along with an allograft carrier, can incorporate and induce bone formation. The MPCs are preimmunodepleted and, therefore, do not stimulate local T-cell proliferation but instead are activated to act as osteoblasts promoting bone formation. As this study is ongoing, no results have been made available.

The therapeutic potential of MPCs has also been reported in the treatment of critical-sized defects of the craniofacial skeleton. Lendeckel et al. reported improved healing in a 7-year-old girl suffering from widespread traumatic calvarial defects, who was treated with autologous adipose-derived MPCs [63]. While CT-scans demonstrated new bone formation, whether the regenerate was a direct outcome of donor cell differentiation or rather a paracrine effect induced by local cells, remains to be elucidated. In another case report, tissue-engineered osteogenic material was injected into a patient undergoing distraction osteogenesis utilizing a fibular flap for mandible reconstruction. The material was comprised of autologous, culture-expanded MPCs that were induced towards osteogenesis via platelet-rich plasma (PRP) activated by thrombin and calcium chloride. Full consolidation of the regenerate was observed after 3 months. Post injection, the regenerated ridge became thicker, and thus aided in bridging a gap between the native mandible and distracted fibula [64]. 

In case reports, other groups have also reported the efficacy of BMSC transplantation in combination with PRP for bone regeneration during distraction osteogenesis [65]. Ueda et al. used this mode of tissue engineering by combining PRP and beta-tricalcium phosphate as grafting materials for maxillary sinus floor augmentation with simultaneous implant placement in 6 patients. A mean increase in mineralized tissue height of 7.3 ± 4.6 mm was evident when comparing the pre- and postsurgical radiographs. Thus, in these cases, injectable tissue-engineered bone provided stable and predictable results in terms of implant success [66].

## 4. Non/Delayed Unions

During normal fracture healing, undifferentiated MPCs are recruited to injury sites and under the influence of regulatory cytokines (e.g., BMPs), and they proliferate and differentiate into chondrocytes and osteoblasts to repair the defect. While many fractures under appropriate conditions heal properly, some fail to heal due to a variety of causes associated with the host, the surgical technique, inadequacy of the vasculature, and infection amongst other causes. At the cellular level, non-union is defined as the cessation of both periosteal and endosteal healing without bridging [67]. Nonunions complicate approximately 10%–20% of 6 million fractures occurring each year in the United States [68] whereas the incidence varies by fracture location. While it remains a common clinical concern, management of nonunions remains a challenge.

In 1978, Salama and Weissman described the first attempt to utilize the osteogenic potential of BMSCs to manage delayed fracture healing in the clinical arena [69]. The authors combined bone xenografts with autologous bone marrow aspirates for grafting in 28 patients for a variety of clinical indications. The treatment of tibial nonunions with autologous marrow injections was expanded further by Connolly et al. who combined this method with either cast immobilization or intramedullary nailing in 20 successful cases over a five-year period [70]. In a followup series of 100 patients, Connolly also employed this technique in the treatment of delayed unions and nonunions of fractures, arthrodeses, and bone defects [71]. Since the original report by Salama et al., numerous researchers have begun utilizing bone marrow aspirates as adjuncts in the treatment of non- or delayed unions with promising results [72].

Hernigou et al. further emphasized the potential role of progenitor cells in bony healing in a study of 60 tibial atrophic nonunions. Using percutaneous injection of a concentrated buffy coat obtained from the centrifugation of autologous iliac crest bone marrow aspirates, the authors noted a positive correlation between the volume of the mineralized callus and the number and concentration of progenitor cells in the aspirate. Analysis of the buffy coat revealed the presence of progenitor cells and other mononuclear cells, most likely providing osteogenic and angiogenic influences. In the 7 patients where union was not accomplished, the concentration of stem cells injected appeared significantly lower than in patients with osseous union (*P* = .001 and *P* < .01). Perhaps a limitation in this study is the lack of a control group with placebo treatment [41]; however, the data demonstrates that successful treatment of nonunion with percutaneous bone marrow grafting is dependent upon the number and concentration of progenitor cells.

In 2005, Goel et al. embarked on a prospective clinical study to evaluate the efficacy of percutaneous bone marrow grafting in 20 patients with established tibial nonunions with minimal deformity, while they waited for open surgical procedures [73]. Three to five milliliters of marrow was aspirated from the iliac crest and injected immediately into and about the site of non-union. Subsequent aspirations were performed 1 cm posterior to the previous site until a maximum of 15 ml of marrow was injected. Clinically and radiologically, bony union was documented in 15 out of 20 patients (75%), with an average time to union following the first injection of 14 weeks. Four patients (20%) showed no evidence of union and were considered a failure. There were no cases of infection following the injections, and no complications at the donor site. Based on these promising results, the authors concluded that percutaneous bone marrow grafting can be considered a safe, simple, and reliable technique for managing non-unions. This minimally invasive method of treating tibial non-unions without deformity can potentially allow the avoidance of major surgical reconstruction in qualified patient populations. 

Maneerit et al. conducted one of the first prospective randomized clinical trials examining the use of bone marrow in the treatment of non- or delayed unions over a 2.5-year period [74]. They compared outcomes between percutaneous bone grafting and open bone grafting of tibial shaft fractures or high-energy tibial fractures which required early prophylactic bone grafts. Subjects were randomized to either percutaneous bone graft (*n* = 15) or open bone graft (*n* = 15). Percutaneous bone graft technique was associated with significantly less blood loss (*P* < .01) and shorter operative time (*P* < .01) although one patient in the percutaneous group had posterior tibial nerve palsy postoperatively with complete recovery after 6 weeks. No differences in rate of union, healing time of the successful cases, postoperative pain and hospital stay were observed, indicating that the percutaneous technique has effective results similar to the open technique in promoting union of tibial fractures. 

Entering the realm of clinical trials marks an important milestone on the pathway to approval of MPC therapy for bony non-unions. In October 2003, Aastrom Biosciences enrolled 36 patients who had failed previous surgical intervention with long-bone atrophic non-unions from type IIIA or IIIb fractures with fracture gaps of <6 cm in a multicenter, nonrandomized, open-label uncontrolled, single-group phase I/II clinical trial (NCT 00424567) [52]. Subjects were treated with open reduction and internal fixation and an allograft bone matrix graft extender plus BMSCs expanded from autologous iliac crest aspirates. In April 2009, the Hadassah Medical Organization in Israel began a randomized, open-label, single-group Phase I/II clinical trial for the treatment of distal tibial fractures (NCT00250302). Twenty-four patients with distal tibial fractures without joint involvement will undergo autologous implantation of MPCs loaded onto carriers at fracture sites to determine the safety and efficacy of this mode of treatment.

## 5. Cartilage Repair

Chondral defects secondary to accidental trauma, necrosis of subchondral bone tissue, or arthritis have become some of the more common conditions today [79]. Approximately 15% of the world's population reportedly suffers from joint diseases. However, despite ongoing research, repair and regeneration of cartilage defects remains a challenge in orthopaedic surgery. Human cartilage is characteristically avascular and depends on diffusion from cyclical loading during joint movement for nutrient acquisition [80]. Given their unique microenvironment, chondrocytes have adapted to a low basic metabolic rate and have limited potential to increase their metabolic activity to allow for tissue repair. As a result, articular cartilage is considered a tissue with minimal intrinsic repair capacity in vivo. 

Currently, many of the treatment modalities including drug therapy, arthroscopy, and prosthetic joint replacement provide symptomatic relief but do not directly address the underlying pathophysiology [81]. Other treatment modalities for cartilage repair, which aim to address the underlying molecular cause, range from bone marrow stimulation, mosaic plasticity, and autologous chondrocyte implantation (ACI). ACI is a method first reported by Brittberg et al. in 1994 [82]. In this technique, chondrocytes are isolated from the cartilage of nonweight bearing sites and expanded ex vivo. These expanded cells are subsequently injected into the defect sites and covered with an autologous periosteal flap to ensure cell adherence. Initial clinical trials of ACI showed promise [83]; however, this treatment requires the extraction of chondrocytes directly from the patient and thus causes trauma to healthy articular cartilage. Other disadvantages of ACI include: leakage of transplanted cells, periosteal hypertrophy, loss of the chondrocyte phenotype in the expanded cells in monolayer culture, lack of applicability to large lesions, and decreased efficacy in patients over 40 years due to low cellular activation levels. Additionally, the newly regenerated cartilage often consists of fibrocartilage, rather than the desired hyaline cartilage within the joint space [84].

Researchers tried to address these initial pitfalls with the development of a second generation ACI procedure. This method employs various biomaterials such as collagen type I gel, hyaluronin-based scaffolds, and collagen type I/III membranes to recreate a 3D environment ideal for expression of chondrogenic phenotypes and to secure cells within the defect site in lieu of a periosteal flap [85–87]. However, prospective clinical studies comparing first and second generation ACI failed to show significant difference in short-term clinical outcomes [88]. 

Although current technologies may improve morbidity associated with local cartilage defects, they still fall short in the treatment of systemic arthritic disease. BMSC therapy for cartilage disorders is principally sound since progenitor cells are typically harvested from the iliac crest, circumventing the need to damage healthy articular cartilage. Furthermore, the number of successfully cultured cells is larger due to their excellent proliferation capacity enabling abundant supply. 

The chondrogenic potential of MPCs was first reported by Ashton et al. in 1980 [89], and since then, many researchers have focused on delineating the mechanism underlying chondrogenic differentiation [90–92]. Currently, only one prospective clinical trial of BMSC transplantation for repair of cartilage defects has been published. Wakitani et al. recruited 24 patients with knee osteoarthritis who underwent a high tibial osteotomy to examine the effects of a cellular versus noncellular impregnated scaffold on cartilage defects in the medial femoral condyle [76]. For the cellular arm, bone marrow-derived MPCs were suspended in a type I collagen gel and transplanted with an autologous periosteal flap into the defect site. The control group also received a periosteal flap but with a cell-free scaffold. No clinically significant improvement was observed in the cell-treated group versus controls, but the arthroscopic and histological scores were higher in the BMSC transplanted subjects. Furthermore, in a later study, the same group investigated the efficacy of autologous BMSCs for repair of cartilage defects and demonstrated clinical and histological improvement in the treated patients [75].

Case reports from the same research group also document improvement in clinical symptoms following BMSC transplantation [93], but comparative clinical trials with other surgical methods must be performed to assess the utility of this type of tissue engineering in humans. Black et al. published the first randomized, double-blind, multicenter control trial examining the effectiveness of stem cell therapy in dogs [94]. They explored the effects of adipose-derived stem cells (ASCs) on lameness in dogs with chronic osteoarthritis of the femoral joint. Dogs treated with ASCs showed significantly improved scores for lameness pain, and range of motion compared with control dogs. While these studies show promising results, it is important to identify the paracrine potential of ASCs and their effect on clinical outcomes. 

Numerous clinical trials are ongoing to examine the utility of MPC therapy in the treatment of cartilage defects. Cairo University School of Medicine has undertaken a Phase II/III clinical trial which began in December 2006 to examine whether implanting autologous, culture-expanded MPCs, obtained from patients with early OA, cartilage defects, or osteochondral joint disease, is effective in treatment of such conditions. Twenty-five subjects will undergo bone marrow aspiration from the iliac crest with implantation of the ex vivo expanded MPCs into defect sites via open surgery or arthroscopy (NCT00891501). Starting in August 2008, The Royan Institute of Tehran University of Medical Sciences enrolled 6 patients in a Phase I study aimed at investigating the efficacy and safety of autologous transplantation of BMSCs mixed with a collagen I scaffold in cartilage defects and osteoarthritis of the knee (NCT00850187). Ullevaal University Hospital in Oslo, Norway began a Phase I clinical trial in April 2009, enrolling 50 patients, to compare the treatment efficacy of autologous MPCs versus chondrocytes implanted in a commercially available scaffold in patients with cartilage defects (NCT00885729). 

In an in vivo study of osteoarthritis in a caprine model, Murphy et al. demonstrated that intra-articular injection of BMPCs at sites of meniscal injury resulted in engraftment of those cells and regeneration of meniscal tissue to produce a chondroprotective effect [95]. Based on results from this in vivo trial, Osiris Therapeutics completed a Phase I/II placebo-controlled, RCT in February 2008 of 60 patients to examine the safety and efficacy of hMPCs suspended in a solution of hyaluronic acid (Chondrogen) for the repair of meniscal tissue following meniscectomy. In Chondrogen treated subjects, surgically removed meniscal tissue was regenerated, cartilage surface was protected, and joint damage was decreased in comparison to control subjects. These benefits persisted for at least one year. As a followup study, in June 2008, Osiris Therapeutics began a second randomized, placebo-controlled Phase I/II clinical trial to examine the long-term safety of high and low-dose BMSCs (Chondrogen) in the repair of meniscal tissue following meniscectomy (NCT00702741). In this 3-year followup study, the treatment group will receive a single intra-articular injection of 50 million donor-derived hMPCs (low dose) or 150 million donor-derived hMPCs (high dose) in suspension with commercial sodium hyaluronan and will be compared to control patients receiving an injection of a vehicle (diluted hyaluronan) alone. 

Although bone marrow provides a good source of stem cells, interest in MPCs derived from the umbilical cord blood (UCB) has recently emerged. Some studies have suggested that the prevalence of MPCs is much higher in preterm UCB as compared to samples obtained from term fetuses [96]. Various studies have suggested that MPCs isolated from UCB have the highest level of activity among all adult stem cells [97]. Using umbilical cord blood for the harvesting progenitor cells also forgoes the ethical implications associated with harvesting embryonic stem cells. With this in mind, in February 2009, Medipost Co Ltd. began a randomized, open-label, multicenter and Phase 3 clinical trial of 104 patients to compare the efficacy and safety of allogeneic-unrelated umbilical cord blood-derived MPC product (Cartistem) to that of a microfracture treatment in patients with articular cartilage defects or injuries (NCT01041001). MPCs were isolated from umbilical cord blood, cultured with semi solid polymer, and surgically administered into lesion sites in order to stimulate the regeneration of defective cartilage tissue.

## 6. Osteonecrosis (ON)

The treatment of osteonecrosis of bone is another arena where cell-based therapies can play a pivotal role. These injected bone marrow cells most likely secrete cytokines that promote angiogenesis and subsequent osteogenesis [98].

Hernigou proposed the use of bone marrow transplantation for treatment of osteonecrosis of the humeral head in 1997 in the case of a 13-year-old patient with sickle cell disease [99]. The donor was an HLA-identical sibling who was heterozygous for sickle cell anemia. Marrow transplantation by intravenous infusion was performed after administration of chemotherapy and total lymphoid irradiation. Three months post transplantation, pain and range of motion was significantly improved, and radiographs revealed rapid reconstruction of the left proximal humerus epiphysis, with a tendency toward normalization of the marrow signal apparent via T1-weighted MR imaging. Thereafter, many clinical studies followed to examine the role of bone marrow aspirates in the treatment of ON in various skeletal locations, demonstrating promising results for the early stages of disease (Table 1) [100]. 

In January 2009, Fuzhou General Hospital in China approved a study to enroll 30 patients in a clinical trial to examine the safety of expanded autologous MPCs infused into the femoral artery of patients with osteonecrosis of the femoral head (NCT00813267). Patients will undergo MPC infusions at the start of the study on day 0 followed by subsequent injections at 6-month intervals. They will be evaluated using digital subtraction angiography, X-ray examination, and CT and MRI scanning.

## 7. Osteogenesis Imperfecta (OI)

OI is a heterogeneous group of inherited disorders characterized by abnormal production of type I collagen by osteoblasts leading to osteopenia, multiple fractures, severe bony deformities and short stature [101–103]. It has variable clinical phenotypes, ranging from subclinical presentation and normal life expectancy to osteopenia or death [104]. Currently, pharmacological management is the treatment of choice. Many studies have demonstrated the utility of bisphosphonate therapy in OI for increasing bone density, strength, and reducing the number of fractures [105]; however, these effects must be confirmed by double blind, randomized control trials. Many researchers also worry about the potential adverse effects of bisphosphonates on the child skeleton, and therefore, raised concern regarding long-term outcomes [106]. 

An alternative treatment is the transplantation of BMSCs, which in principle should alleviate or resolve a genetic disorder of bone. In fact, preclinical experiments carried out in animal models revealed that transplanted marrow stromal cells can migrate and incorporate into bone of recipient animals [107]. Horwitz et al. have been utilizing allogeneic bone marrow transplantation for the treatment of severe OI in children since 1999 [108]. In their latest case report, the authors described the use of gene-marked, donor marrow-derived mesenchymal cells to treat 6 children who underwent standard bone marrow transplantation for severe OI. Each child received two infusions of the allogeneic cells. One patient developed an urticarial rash immediately following the second infusion, but otherwise, no clinically significant toxicity was reported [78]. 

Taking the concept of bone marrow transplantation for OI one step further, Le Blanc and colleagues performed an intrauterine transplantation of a female fetus diagnosed with severe OI at 32 weeks of gestation with MPCs isolated from a HLA-mismatched male fetus [77]. At 9 months of age, bone histology showed regularly arranged and configured bone trabeculae. Patient lymphocyte proliferation against donor MPC was not observed in coculture experiments performed in vitro after MPC injection. During the first 2 years of life, three fractures were noted, and at 2 years of corrected age, psychomotor development was normal, and growth followed the same channel. Given these findings, the authors concluded that allogeneic fetal MPCs can engraft and differentiate into bone in a human fetus even when the recipient is immunocompetent and HLA-incompatible. 

Encouraged by previous clinical experiences, Horwitz et al. have begun a Phase I, nonrandomized control trial of 12 patients with Type II or III OI in order to evaluate the safety and effectiveness of repeated BMSC infusions (NCT01061099). Group A will consist of patients who have previously undergone bone marrow transplant whereas those in Cohort B lack a history of bone marrow transplantation. All participants will receive BMSC infusions approximately every 4 months to complete a total of 6 infusions over a 20-month period and will be followed for 4 months after their last infusion.

Drexel and Wayne State University underwent a Phase I pilot study from July 1999 to January 2008 of 8 patients with osteodysplasia who had undergone a previous bone marrow transplantation (NCT00186914). They were infused twice with ex vivo expanded, allergenic, gene marked donor BMSCs. The first dose was given at least 6 months post transplant and the second at 14 to 21 days after. Since the stromal cells were obtained from the original stem cell donor, no conditioning was required. Study results are still pending.

## 8. Tendon Repair

Currently, experimental animal models are in progress to establish convincing evidence for enhanced tendon repair with progenitor cell therapy, but advancements in the field have not yet reached the level of clinical trials. Investigations to date have demonstrated the utility of Type I collagen combined with autologous, expanded BMSCs for improving the biomechanical properties of injured rabbit tendons although differences in microstructure have yet to be seen [109]. 

Using an equine model, Crovace et al. injected and compared cultured bone marrow mesenchymal stem cells (cBMSCs) and bone marrow mononucleated cells (BMMNCs) versus placebo for treatment of collagenase-induced tendonitis in the horse. At 21 weeks, histological analysis and immunohistochemical stains with H&E and Herovici for collagen type I and III revealed mature type I collagen with normal architecture in tendons treated with cBMSC and BMMNC, while random collagen type III organization was observed in the placebo group. These results suggest that cBMSC and BMMNCs have the potential to promote tendon regeneration in an equine collagenase-induced tendonitis model [110]. Authors have also used BMSCs seeded onto various scaffolds and found that introducing autogenous mesenchymal progenitor cells onto composites significantly improved tendon repair compared to the use of a composites alone in the rabbit model [111]. 

One potential pitfall of BMSC therapy for tendon repair is the potential for ectopic bone production at the site of injury. Harris et al. observed this phenomenon in 28% of BMSC-treated rabbit tendons and concluded that better control of the differentiation pathway with additional in vitro testing is necessary prior to embarking on clinical trials with MPC therapy in tendon repair [112].

## 9. The Future of Stem Cells

Much attention has been engendered for combining the principles of stem cell therapy with those of gene therapy to engineer cells that can complement cellular function in genetic disorders, as discussed previously. Gene therapy can be executed ex vivo with the gene of interest introduced to the progenitor cells followed by its readministration back into the patient, thereby replacing the missing factor in the host. To date, these approaches have only been studied in animals but with great success. 

Prior to embarking on clinical trials, the vectors used to deliver genes of interest must be optimized. Recombinant forms of bone morphogenic proteins (rhBMPs), for example, have been used in animal models to promote and hasten osteogenesis [113]. Based partly on this in vivo work, the US Food and Drug Administration approved the use of the rhBMP-2 and rhBMP-7 in spinal fusions and tibial non-unions, respectively. Although promising and seemingly effective, rhBMPs have multiple disadvantages, namely, the requirement of supraphysiologic concentrations and low biological activity due to high rates of clearance from the defect sites [114]. In addition, high costs and difficulty of production are potential factors limiting the use of rhBMPs in clinical practice. 

An alternative mode of applying BMPs is through their expression using adenoviral vectors. This form of gene therapy is able to deliver recombinant BMP DNA to cells at the defect sites [115]. Treated cells can then synthesize and secrete their own endogenous BMPs and supply the extracellular environment with a continuous concentration of osteoinductive signaling factors without the need of reapplication [114]. These adenoviral vectors provide a short but high level expression of the gene of interest, which is sufficient to promote osteoblastic differentiation and subsequent bone formation [116]. Baltzer et al. used adenoviruses expressing BMP-2 (AdBMP-2) to induce healing of critical-sized bone defects in rat femurs [117], and other investigators have followed suit [118]. Our laboratory focuses in particular on the role of adBMP-9 in osteogenic differentiation of mesenchymal progenitors [119]. 

However, in vivo transfer of genes utilizing viral vectors provokes the possibility of immune reaction, which will prevent treatment efficacy. And thus, because of the strong potential for adverse reaction, utilizing adenovirus in human trials remains distant. Current strategies underway involve ex vivo genetic modification of autologous BMSCs via adenoviral vectors followed by their reimplantation in vivo. This approach avoids the transfer of viral particles or DNA directly into the patient and would most likely forgo the immune response associated with direct viral administration [120]. To date, however, ex vivo adenoviral gene therapy has been utilized only in animal models.

Concern surrounding the use of viral vectors as vehicles for gene delivery has prompted the utilization of other methods to genetically modify MPCs. Nucleofection is a modified electroporation technique that has been used by many different groups for genetic modification of BMSCs [121–123]. FuGENE 6, a cationic polymer-based commercially available transfection reagent, has also been successfully used to genetically engineer MPCs [124–126]. In addition, liposome-mediated transfection of MPCs has also been reported [127]. While these novel nonviral methods of transfecting cells are promising, significant research is necessary to delineate their effectiveness. 

Genetic engineering of BMSCs is ideal, as it eliminates the requirement of large amounts of cells for implantation and culture expansion. Although the morbidity associated with bone marrow aspiration for the accrual of MPCs is less than previous methods of defect repair that utilized autologous bone grafting, researchers remain interested in developing more minimally invasive means of harvesting progenitor cells. One such modality which has gained popularity is utilizing adipose tissue as a source of progenitor cells. Given the ease of harvest and the availability of adipose tissue, many researchers are currently interested in understanding the mechanisms underlying the differentiation capacity of adipose-derived progenitor cells (APCs) [128]. While adipose-derived MPCs show significant promise as an important source of MPCs, further research into their characteristics as progenitor cells is necessary to harness their utility in the clinical setting. 

## 10. Conclusion

The therapeutic capacity and safety of BMSCs have been documented in numerous animal experiments in vivo. Currently, 107 clinical trials utilizing exogenous BMSCs to treat a wide range of conditions are registered with ClinicalTrials.gov (Tables 2 and 3). Although many are also underway to examine the role BMSCs in orthopaedic associated tissue regeneration, limited evidence is currently available to support routine use. Results from large-scale multicenter clinical trials must be completed and analyzed prior to reaching the final destination of FDA approval for utilizing cell-based therapy to manage orthopaedic patients in the clinic. Given the abundance of ongoing investigations, however, we can expect a profuse amount of new clinical data in the near future. Science has progressed infinitely since Friedenstein's pioneering studies in the 1960s, and with the continuation of human trials, we move one step closer towards applying BMSC therapy as a novel paradigm.

## Figures and Tables

**Table 1:** Clinical studies of bone marrow aspirates in osteonecrosis.

Authors	Year	Study	Description	Main findings
Hernigouet al.	1997	Case report	13-year-old with SSA and ON of humeral head treated with allogenic bone marrow transplantaion	Improvement in pain and motion range; X-Rays at 3 months showed significant regeneration of the proximal humerus and epiphysis

Hernigouet al.	2002 2005	Prospective noncontrol	189 hips in 116 patients were treated with core decompression and with ABM harvested from anterior iliac crests; the aspirated marrow was reduced in volume by concentration and injected into the femoral head after core decompression with a small trocar; followup of 5–10 years	In 145 of the stage I/II patients, hip replacement was required in 9; total hip replacement was necessary in 25 of 44 hips operated on at stage III/IV patients with greater number of OPGr cells transplanted had better outcomes

Gangi et al.	2002	Prospective control	Treatment of 18 hips with stage 1 or 2 (ARCO) femoral head ON with either core decompression alone or in combination with ABM injection	Significant reduction in pain and joint symptoms at 2 years; 5 of 8 hips in control group deteriorated to stage 3 compared to 1 hip in treatment arm

Yan et al.	2006	Prospective noncontrol	Treatment of 44 hips with stage 3 or 4 (ARCO) femoral head ON with ABM injection	Mean Harris hip score improved from 58 (46–89) to 86 (70–94) at 2 years; no complications

Kawate et al.	2006	Case reports	Treatment of 3 patients with steroid-induced ON of femoral head (ARCO Stages 4A–4C) by transplantation of autologous MSCs cultured with beta-tricaclium phosphate ceramics and free vascularized fibulas	Early bone regeneration observed but radiographic progression was seen in 2 of 3 patients at mean followup of 34 months

Lee et al.	2009	Case reports	Three patients with bilateral, large lesions (0.32 of femoral width) of ON of the femoral condyles treated by decompression, debridement, and ABM grafting using the Cellect DBM System to increase the number of OPGr cells from the aspirate	Cellect provided graft matrix enriched with a 3-fold to 4-fold increase in OPGr cells; no complications at 2 years with all 3 patients achieving near-normal function and activity levels

ABM: autologous bone marrow; ARCOL: association research circulation osseous; OPGr: osteoprogenitor; SSA: sickle cell anemia.

**Table 2:** Examples of the therapeutic applications of MPCs in humans.

Indications	Source	Mode of administration	Outcome
Fracture nonunion	Autologous BM	100% hydroxyapatite macroporous ceramic scaffolds with MPCs	X-ray & CT evidence of bone formation: recovered limb function [60]
	Autologous BM	Subcutaneous	Correlation between volume of mineralized callus and concentration of progenitor cells in the aspirate [40]

Cartilage defect	Autologous BM	Direct site transplantation	Improved clinical symptom and coverage of defect [75]
	Autologous BM	Cells embedded in collagen gel transplanted at site of cartilage defect	Improvement in arthroscopic and histologic grading [76]

Osteogensis imperfecta	Fetal MSC	Intrauterine transplantation	Osteoblastic differentiation and reduced fracture [77]
	Gene-marked Allogenic MPCs	IV infusion × 2	5 out of 6 patients demonstrated bone engraftment and increase in bone velocity [78]

Critical size defect	Autologous BM	Scaffold loaded	Faster full recovery of limb function than bone graft [59]

Craniofacial defect	Autologous Adipose-derived MPCs	Local administration of cells with fibrin glue	CT scans showed new bone formation and near complete calvarial continuity 3 months postoperatively [63]

MPC: mesenchymal progenitor cell, BM: bon marrow, IV: intravenous.

**Table 3:** Ongoing progenitor cell-therapy in orthopaedic patients.

Indications	Sponsors	Phase	Age range	Study type	Intervention	Source	Route of administration	Clinical TrialID no.	Status
Bone defects	Emory University	II + III	11 years and older	Interventional	Trinity MPC & DBM	Commercial	Direct filling of bone defects with progenitor cells	NCT00851162	Not yet recruiting

Fracture nonunion	Aastrom Biosciences	I + II	18 years and older	Interventional	Fracture surgery + Cultured BM tissue	Autologous BM	Direct admiministration to site of fracture	NCT00424567	Completed

Dital tibial fracture	Hadassah Medical Organization	I + II	18–65 years	Interventional	Autologous “MSC” implantation	Autologous BM	Cells loaded onto a carrier and implanted locally at the fracture site	NCT00250302	Recruiting

Knee Cartilage defects osteoarthritis	Royan Institute	I	45–60 years	Interventional	Autologous “MSC” implantation	Autologous BM	Cells loaded on collagen I scaffold are implanted locally to the cartilage defect site	NCT00850187	Recruiting

Osteochondral defects	Cairo University	I + II	15–55 years	Interventional	Autologous “MSC” implantation	Autologous BM	Cell pellets will be implanted into osteochondral defect via open surgery or arthroscopically	NCT00891501	Recruiting

Cartilage Defects	Ullevaal University Hospital	I	18–50	Interventional	Autologous “MSC” implantation versus chondrocyte implantation	Commercial	Cells loaded on commercially available scaffolds and implanted locally at the cartilage defect site	NCT00885729	Recruiting

Meniscectomy	Osiris Therapeutics	I + II	18–60	Interventional	Chondrogen versus placebo	Chondrogen (commercial)	Intra-articular injection	NCT00702741	Ongoing but not recruiting

Chondral defect	Medipost Co Ltd.	III	18 years and older	Interventional	Cell therapy versus microfracture	UCB	Culture expanded cells mix semisolid polymer will be implanted locally at the site of chondral defect	NCT01041001	Recruiting
Osteonecrosis of the femoral head	Fuzhou General Hospital	I + II	12–60 years old	Interventional	Autologous MSC	N/A	Direct cellular infusion through tubes inserted into MFCA, LFCA, and OA	NCT00813267	Not yet recruiting

OI Type I + II	Children's Hospital of Philadelphia	I	≤19 years	Interventional	Infusion Haploidentical MSCs in patients with history of VM transplant versus No history of transplant	Donor BM	Repeat infusion of MSCs in sub	NCT01061099	Recruiting

Osteodysplasia	St. Jude Children's Research Hospital	I	N/A	Interventional	Infusion of ex vivo expanded gene marked BMSC following allogenic BM transplantation	Donor BM	IV Infusion	NCT00186914	Completed

MPC: mesenchymal progenitor cells, DBM: demeneralized bone matrix, BM: bone marrow, MSC: mesenchymal stem cell, MFCA: medial femoral circumflex artery, LFCA: lateral femoral circumflex artery, OA: obturator artery, IV: intravenous, UCB: umlical cord blood, N/A: not available.
